# The Urokinase Receptor Takes Control of Cell Migration by Recruiting Integrins and FPR1 on the Cell Surface

**DOI:** 10.1371/journal.pone.0086352

**Published:** 2014-01-21

**Authors:** Anna Gorrasi, Anna Li Santi, Giuseppina Amodio, Daniela Alfano, Paolo Remondelli, Nunzia Montuori, Pia Ragno

**Affiliations:** 1 Department of Chemistry and Biology, University of Salerno, Salerno, Italy; 2 Department of Medicine and Surgery, University of Salerno, Salerno, Italy; 3 Department of Translational Medical Sciences, “Federico II” University, Naples, Italy; University of Birmingham, United Kingdom

## Abstract

The receptor (uPAR) of the urokinase-type plasminogen activator (uPA) is crucial in cell migration since it concentrates uPA proteolytic activity at the cell surface, binds vitronectin and associates to integrins. uPAR cross-talk with receptors for the formylated peptide fMLF (fMLF-Rs) has been reported; however, cell-surface uPAR association to fMLF-Rs on the cell membrane has never been explored in detail.

We now show that uPAR co-localizes at the cell-surface and co-immunoprecipitates with the high-affinity fMLF-R, FPR1, in uPAR-transfected HEK-293 (uPAR-293) cells. uPAR/β1 integrin and FPR1/β1 integrin co-localization was also observed. Serum or the WKYMVm peptide (W Pep), a FPR1 ligand, strongly increased all observed co-localizations in uPAR-293 cells, including FPR1/β1 integrin co-localization. By contrast, a low FPR1/β1 integrin co-localization was observed in uPAR-negative vector-transfected HEK-293 (V-293) cells, that was not increased by serum or W Pep stimulations.

The role of uPAR interactions in cell migration was then explored. Both uPAR-293 and V-293 control cells efficiently migrated toward serum or purified EGF. However, cell treatments impairing uPAR interactions with fMLF-Rs or integrins, or inhibiting specific cell-signaling mediators abrogated uPAR-293 cell migration, without exerting any effect on V-293 control cells.

Accordingly, uPAR depletion by a uPAR-targeting siRNA or uPAR blocking with an anti-uPAR polyclonal antibody in cells constitutively expressing high uPAR levels totally impaired their migration toward serum.

Altogether, these results suggest that both uPAR-positive and uPAR-negative cells are able to migrate toward serum; however, uPAR expression renders cell migration totally and irreversibly uPAR-dependent, since it is completely inhibited by uPAR blocking.

We propose that uPAR takes control of cell migration by recruiting fMLF-Rs and β1 integrins, thus promoting their co-localization at the cell-surface and driving pro-migratory signaling pathways.

## Introduction

To reach their final destination or their workplace, cells must move through the extracellular matrix (ECM) and, sometimes, also between each other. Cell migration is essential for many biologic and pathologic processes and is the result of highly coordinated events which involve cell polarization, actin-driven protrusion, formation and turn-over of cell adhesions, localized ECM degradation [1].

Since many years, the receptor (uPAR) of the urokinase-type plasminogen activator (uPA) serine-protease has been considered crucial in cell migration processes since it concentrates uPA proteolytic activity at the cell surface, thus allowing localized ECM degradation [2]. Indeed, uPAR is moderately expressed in various tissues in the healthy organism, but its expression strongly increases in organs undergoing extensive tissue remodeling. uPAR expression is also increased in many pathologic conditions, in particular in cancer, inflammation and infections [2]–[3].

uPAR is a heavily glycosylated protein formed by three cysteine-rich LY6-like domains (DI, DII, and DIII, from the external N-terminus) connected by short linker regions. It is anchored to the cell surface through the glycosyl-phosphatidylinositol (GPI) tail of the C-terminal DIII. The three uPAR domains define a deep cavity which accomodates uPA, leaving the whole external surface available for other potential interactions [4]. Indeed, uPAR acts also as a high affinity receptor for vitronectin (VN), an ECM component, particularly abundant in ECM associated to tumor tissues [5]. Both uPA and VN, which require full-length uPAR for binding, are able to activate intracellular signaling pathways, leading to cell proliferation, survival, adhesion and migration, in spite of the absence of a transmembrane and a cytosolic region in the uPAR molecule [6]. Thus, cell surface molecules, able to associate to uPAR and to connect uPAR to intracellular signaling pathways, have been largely investigated. Integrins seem the most probable candidates as uPAR signaling partners [7]. In fact, uPAR-integrin association has been shown by co-immunolocalization, co-immunoprecipitation, FRET and by *in vitro* binding assays between purified uPAR and α5β1 integrin [8]. Despite the controversy surrounding whether uPAR and integrins interact physically, a large body of evidence shows that uPAR signaling requires integrins as co-receptors. uPAR, beside using integrins, also regulates their activity, with different extents in different cell systems [8].

The linker region between the N-terminal DI and DII uPAR domains is extremely sensitive to various proteases, including uPA; the proteolytic cleavage removes DI and generates a shorter uPAR form (DIIDIII-uPAR), unable to bind both uPA and VN and to associate to integrins [9].

Both full-length and cleaved uPAR can be released by the cell surface in soluble forms. The soluble form of DIIDIII-uPAR (s-DIIDIII-uPAR) exposing the SRSRY sequence (aa 88–92) at its N-terminus, is unable to bind both uPA and VN, as its cell-membrane counterpart, nevertheless it acquires a new important activity; in fact, it is a ligand for the G-protein-coupled receptors for the fMLF (fMet-Leu-Phe) peptide, a peptide of bacterial origin [9]–[10].

Three fMLF receptors (fMLF-Rs) have been identified and cloned: the high-affinity *N*-formyl-peptide receptor (FPR1) and its homologue FPR-like 1 (FPR2) and FPR-like2 (FPR3) receptors. FPR2 has a much lower affinity for fMLF, but it is efficiently activated by several other molecules, including lipoxin A_4_, serum amyloid A, HIV derived peptides. FPR3 shows a high homology with the other two fMLF-Rs but does not bind fMLF and shares few ligands with the other fMLF-Rs [11]. Activation of fMLF-Rs by their ligands induces cell migration. Thus, s-DIIDIII-uPAR is a potent chemoattractant for cells expressing FPR1, FPR2 or FPR3 [2]. The SRSRY sequence that, in the soluble uPAR form, is unmasked only after the removal of DI, is instead exposed in the cell-anchored receptor, as demonstrated by the observation that an antibody directed against this specific uPAR region (residues 84–95, uPAR_84–95_) reacts with full-length cell-surface uPAR and does not with the full-length soluble form (suPAR) [12]. It is then reasonable to hypothesize that also full-length cell-surface uPAR could be able to interact with fMLF-Rs through the same sequence of the cleaved suPAR. However, GPI-uPAR co-localization or association to fMLF-Rs on the cell membrane has never been explored in detail.

A high number of cell-surface molecules interacting with uPAR have been reported, including most of integrin families, growth-factor receptors as the receptors for the epidermal growth factor (EGFR) and for the platelet derived growth factor receptor (PDGFR)-beta, and several other molecules [13]–[14]. Thus, uPAR seems to interact with a multitude of different molecules with disparate functions, using some of them as signaling partners and/or regulating their activity.

Indeed, it would be reasonable that only few and specific molecules could really interact with uPAR, thus regulating its relations with the other neighbour cell-surface molecules. In fact, we recently showed that uPAR is able to regulate the activity of the receptor for the stromal derived factor 1 (SDF1) chemokine, CXCR4, in a fMLF-R- and integrin-dependent manner [15]; this finding is greatly in agreement with results by Furlan et al., which showed that the cleaved form of soluble uPAR can modulate the ability of monocytes to migrate toward MCP-1 and RANTES chemokines by binding FPR2 and decreasing chemokine-induced integrin-dependent rapid cell adhesion [16]. These evidences would suggest that integrins and fMLF-Rs could represent possible functional intermediators between uPAR and the other cell-surface molecules.

On this basis, we aimed firstly to assess uPAR/fMLF-R localization and association at the cell surface and to evaluate the relationship of this potential complex with integrins. Then, we intended to explore the role of GPI-uPAR interactions with fMLF-Rs and integrins in regulating uPAR cross-talk with other cell-surface receptors, by evaluating the effects of such interactions on cell migration to serum, which contains various and different chemoattractants able to bind various and different cellular receptors.

## Materials and Methods

### Reagents

The rabbit anti-uPAR polyclonal 399 antibody was from American Diagnostica (Greenwich, CT); the anti-uPAR monoclonal antibodies R2 and R4 were kindly provided by Dr. G. Hoyer-Hansen (Finsen Laboratory, Copenhagen, Denmark). Mouse monoclonal antibodies against β1 integrins and FPR2, and rabbit polyclonal antibodies against β1 integrins, FPR1 and FPR3 were from Santa Cruz Biotechnology (Santa Cruz, CA). Rabbit anti-β-actin, mouse anti-tubulin antibodies, mouse anti-FLAG M2, uPAR-targeting and control siRNAs, the protease inhibitor cocktail and the inhibitors of PI-3K and ERKs were from SIGMA (St. Louis, MO). Inhibitors of the Rac-specific GEF (guanine nucleotide exchange factor) Trio and Tiam1 and of the Rho-associated kinase (ROCK) were from Calbiochem (Darmstadt, Germany). The rabbit antibody recognizing the SRSRY sequence of uPAR has been developed by PRIMM (Milan, Italy) by using the uPAR_84–95_ peptide (corresponding to uPAR residues 84–95, which include the SRSRY sequence) assembled onto a branching lysine core (15). EGFP- tagged uPAR, inserted in the pEGFP-N1 vector, was a kind gift of Dr. N. Sidenius (IFOM, Milan, Italy), and fMLF-R cDNAs were kindly provided by Dr. M. Perretti (William Harvey Research Institute, London, United Kingdom). Cy3 or Alexa 488 conjugated secondary antibodies were purchased from Jackson Immunoresearch (West Grove, PA) and the Prolong AntiFade kit and Lipofectamine 2000 from Invitrogen (Grand Island, NY). Horseradish peroxidase-conjugated anti-mouse and anti-rabbit IgG were from Bio-Rad (Hercules, CA). ECL detection kit was from Amersham International (Amersham, England) and Polyvinylidene fluoride (PVDF) filters from Millipore (Windsor, MA). Collagen was purchased from Collaborative Research (Bedford, MA) and the chemotaxis polyvinylpyrrolidone-free (PVPF) filters from Whatman Int. (Kent, UK). The W (WKYMVm) and the P-25 (AESTYHHLSLGYMYTLN) [17] peptides were synthesized by PRIMM (Milan, Italy).

### Cell culture

Human embrional kidney 293 (HEK-293) and prostate carcinoma (PC3) cells were grown in DMEM supplemented with 10% foetal bovine serum (FBS). Stably transfected cells were grown in DMEM additioned of 10% FBS and 0.5 mg/ml Geneticin.

### Transfections

uPAR-293 cells are HEK-293 previously transfected with the entire coding region of uPAR cDNA cloned in the EcoRI site of the pcDNA3 vector [18]. These cells were transiently transfected with FPR1, FPR2 or FPR3 cDNAs inserted in a pcDNA3 vector for co-immunoprecipitation assays or with FPR1 cDNA for Immunofluorescence analysis. EGFP-tagged uPAR, cloned in the pEGFP-N1 vector [19], or the empty vector, were transiently transfected in HEK-293 cells, which are uPAR negative cells [17], for Immunofluorescence analysis. In both cases, 2.5×10^6^ cells, plated in 60 mm dishes, were transfected with 9 µg of DNA and 22.5 µl of LipofectAMINE 2000 (Invitrogen) in serum-free DMEM for 5 h at 37°C (5% CO2). Cells where then lysed after 48 h for co-immunoprecipitation experiments or treated for the Immunofluorescence analysis.

To knock-down uPAR, 2×10^5^ PC3 cells were seeded in 35 mm plates and transfected with 100 nM uPAR-targeting or control siRNAs in antibiotic-free medium using Oligofectamine, according to the manufacturer's instructions. Cells were incubated for 48 h at 37°C, 5% CO2, and then washed and lysed in 1% TX-100 or loaded in Boyden chamber for migration assays.

### Immunofluorescence analysis and co-localization quantification

uPAR-293 cells, grown on glass coverslips and transiently transfected with FPR1 cDNA, or HEK-293 cells, grown on glass coverslips and transiently transfected with EGFP-uPAR or the pEGFP-N1 empty vector, or with the 3×FLAG-Frizzled-4(fz4) receptor cDNA as co-localization control, were washed and fixed 10 min in 4% paraformaldehyde. Then, FPR1-transfected uPAR-293 cells were incubated for 1 h at room temperature with 4 µg/ml of R4 anti-uPAR monoclonal antibody and 2 µg/ml of FPR1-specific rabbit polyclonal antibody, or with 4 µg/ml of R4 and 4 µg/ml of β1 integrin-specific rabbit polyclonal antibody; HEK-293 cells transfected with EGFP-uPAR or the pEGFP-N1 vector were incubated for 1 h at room temperature with 2 µg/ml of FPR1-specific rabbit polyclonal antibody and 4 µg/ml of anti-β1 integrin mouse monoclonal antibody; HEK-293 cells transfected with 3×FLAG-fz4 were incubated for 1 h at room temperature with 0.5 µg/ml of mouse anti-FLAG M2 and 2 µg/ml of FPR1-specific rabbit polyclonal antibody or with mouse anti-FLAG M2 and 4 µg/ml of anti-β1 integrin rabbit antibody. Then, uPAR-293 cells or 3×FLAG-fz4-HEK-293 cells were further incubated with Cy3 or Alexa 488 conjugated secondary antibodies, and EGFP-uPAR-293 cells or their negative control with Alexa 594 and Cy5 conjugated secondary antibodies.

Coverslips were mounted with the Prolong AntiFade kit. Images were collected as specified using a laser scanning confocal microscope (LSM 510; Carl Zeiss MicroImaging) equipped with plan Apo 63×, NA 1.4 oil immersion objective lens. The percent of co-localization of the fluorescence signals was calculated multiplying by 100 the Manders co-localizing coefficients, as measured by the LSM 510 4.0 SP2 software. In detail, 8 bit images are acquired and subjected to intensity threshold in order to eliminate background intensities. The threshold was set to 100 for every image analyzed and the percent of co-localization was quantified on a minimum of 50 different cells. The percent of co-localization is relative to the single z-plane stack shown in the immunofluorescence panels; no significant differences of co-localization were found in the range of 0.5 µm above or below the shown in-focus z-plane.

### Co-immunoprecipitation

Immunoprecipitation was performed as previously described [17]. The cells were washed twice with microtubule stabilization buffer (0.1 M Pipes, pH 6.9, 2 M glycerol, 1 mM EGTA, 1 mM magnesium acetate) and then extracted in 0.2% Triton X-100 in the presence of protease inhibitors. The insoluble residue, enriched in cytoskeleton-associated proteins, was solubilized in RIPA buffer (150 mM NaCl, 50 mM Tris-HCl, pH 7.5, 1% deoxycholate, 0.1% SDS, 1% Triton-X 100, and protease inhibitors) and preincubated with nonimmune serum and 10% protein A-Sepharose for 2 h at 4°C. After centrifugation, the protein content of the supernatants was measured by a colorimetric assay (BIORAD) and 0.5 mg of protein was incubated for 2 h at 4°C with 30 µg/ml of rabbit polyclonal antibodies against FPR1 or FPR3 or of a mouse monoclonal antibody directed to FPR2, or with 30 µg/ml of nonimmune rabbit or mouse immunoglobulins, as controls. After 30 min of incubation with 10% protein A-Sepharose at room temperature, the immunoprecipitates were washed, subjected to 10% SDS-PAGE, and analyzed by Western blot using 1 µg/ml of the R2 anti-uPAR monoclonal antibody for FPR1 and FPR3 immunoprecipitates, or 2 µg/ml of the 399 rabbit polyclonal antibody for FPR2 immunoprecipitates. Finally, washed filters were incubated with horseradish peroxidase-conjugated secondary antibodies and detected by ECL.

### Cell migration assay

Cell migration assays were performed in Boyden chambers using 8 µm pore size PVPF polycarbonate filters coated with 50 µg/ml of collagen. 2×10^5^ transfected-HEK-293 cells or 1×10^5^ PC3 cells were loaded in the upper chamber in serum-free medium; 10% FBS-DMEM or 100 ng/ml EGF were added in the lower chamber as chemoattractants. Transfected-HEK 293 cells and PC3 cells were allowed to migrate for 4 h and 2 h, respectively, at 37°C, 5% CO2. Then, the cells on the lower surface of the filter were fixed in ethanol, stained with hematoxylin, and counted at 200× magnification (10 random fields/filter). When indicated, cells were preincubated with 5 µg/ml of polyclonal antibodies directed to full-length uPAR or uPAR_84–95_ region, or with 50 µM of P-25 peptide for 1 h at room temperature, or with 10 µM of inhibitor of Rho-signaling or 50 µM of inhibitor of Rac-signaling or 10 µM of ERK inhibitor or 20 µM of PI3K inhibitor for 30 min at 37°C, or with 5 nM of W peptide for 1 h at 37°C, 5% CO2.

### Statistical analysis

Differences between each group of values and its control group were evaluated by the Student's t test using PRISM software (GraphPad, San Diego, CA). *P*≤0.05 was considered statistically significant.

## Results

### uPAR and FPR1 co-localize at the surface of uPAR-transfected HEK-293 cells

**Figure 1:**
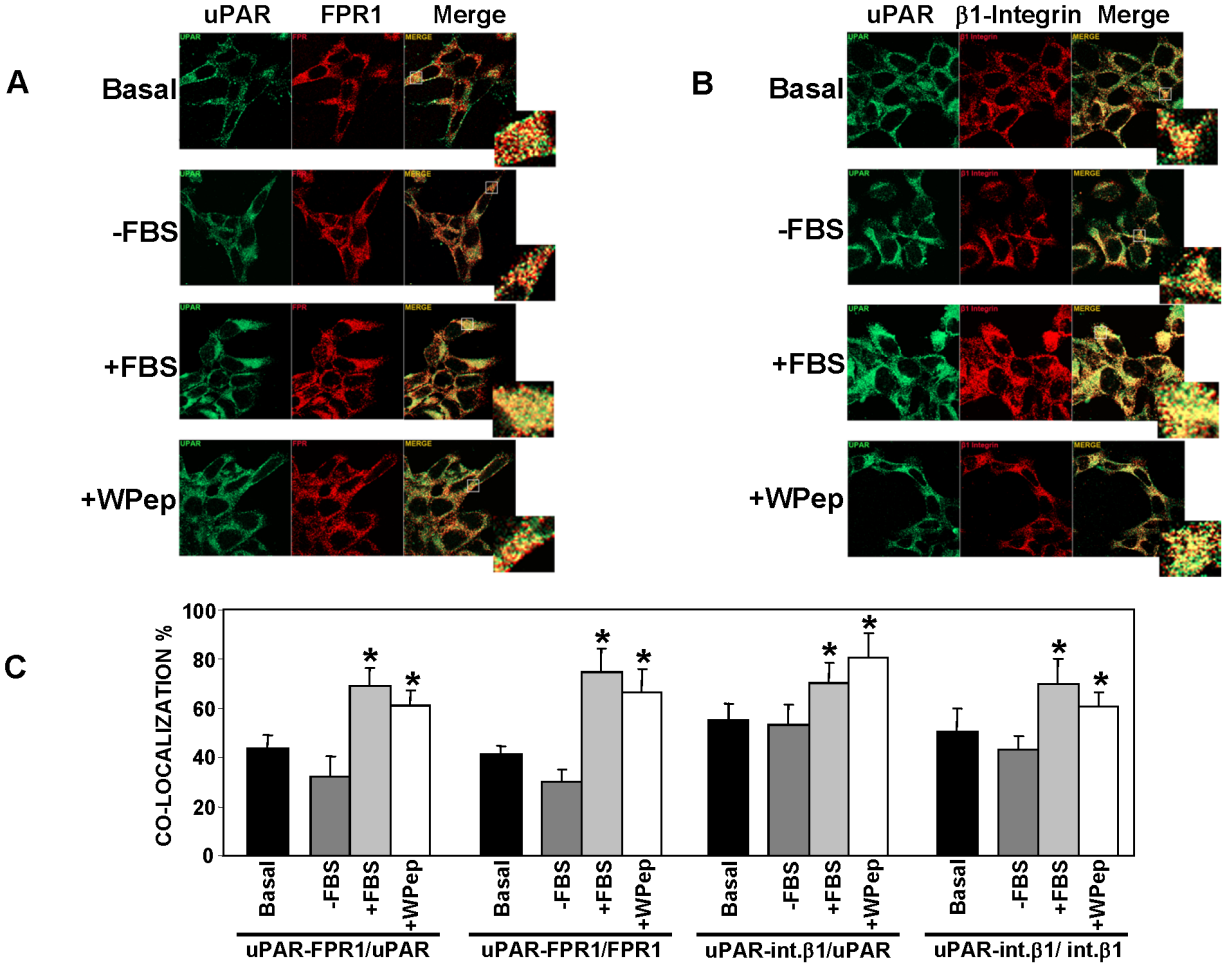
FPR1 co-localizes with uPAR on the surface of uPAR-expressing HEK-293 cells. HEK-293 cells stably transfected with uPAR cDNA (uPAR-293 cells) were seeded on glass coverslips and transiently transfected with FPR1 cDNA. After 24 h, cells were incubated for further 24 h in culture medium (DMEM) containing 10% serum (Basal) or in DMEM without serum (−FBS). Prior to fixation, some serum-starved samples were stimulated for 1 h at 37°C, 5% CO2, with 10% FBS in DMEM (+FBS) or with 5 nM WKYMVm peptide (+W Pep). All samples were then fixed, incubated with the anti-uPAR monoclonal R4 antibody and the anti-FPR1 polyclonal antibody (**A**) or with the anti-uPAR monoclonal R4 antibody and the rabbit anti-β1 polyclonal antibody (**B**), stained with Cy3 or Alexa 488 conjugated secondary antibodies, and analyzed by confocal microscopy. The insets show a 4× magnification for the indicated region in each merge image. **C:** The degree of co-localization of the fluorescent signals was quantified on a minimum of 50 different cells by using the LSM 510 software. The number of co-localizing pixels was normalized to the total pixels of each fluorophore. Thus, the number of yellow pixels, corresponding to co-localizing uPAR-FPR1, has been normalized to green (uPAR) or red (FPR1) pixels shown in A and reported in 1^st^ and 2^nd^ set of columns, respectively. The number of pixels corresponding to co-localizing uPAR-β1 integrin has been normalized to green (uPAR) or red (β1 Integrin) pixels shown in B and reported in 3^rd^ and 4^th^ set of columns, respectively. (*) p≤0.05, as determined by the Student's *t* test.

We and others previously observed a cross-talk between cell-anchored uPAR and fMLF-Rs [18], [20]–[22]; however, their association on the cell membrane has never been explored in detail. Thus, we firstly investigated uPAR and fMLF-R co-localization on the cell surface. To this end, we used uPAR-negative HEK-293 cells [5], [17]–[18], stably transfected with uPAR cDNA [18] and here named uPAR-293. Since HEK-293 cells mainly express FPR1 [18], we focused our studies on uPAR association to this fMLF-R. uPAR-293 cells were transiently transfected with the FPR1 cDNA, to reinforce FPR1 expression, and analyzed by confocal microscopy with uPAR- and FPR1-specific antibodies; the analysis confirmed the expression of both receptors on the cell surface and showed their co-localization (Fig. 1A, top panel). Quantification of uPAR-FPR1 co-localization, obtained by normalizing the pixels corresponding to co-localizing uPAR-FPR1 (yellow) to total uPAR pixels (green) or to total FPR1 pixels (red), showed that, in basal conditions, about 44% of uPAR co-localized with FPR1 and about 41% of FPR1 co-localized with uPAR on the cell surface of transfected cells (Fig. 1C, first and second set of columns).

To investigate whether external stimuli can increase such uPAR/FPR1 co-localization, transfected cells were serum-starved for 24 h and then incubated for 1 h with 10% serum or with a fMLF-R ligand, the W Peptide (WKYMVm; W Pep) [23]. In the absence of serum, uPAR/FPR1 co-localization values were lower than those in basal conditions, however, cell stimulation with serum or with the W Pep, strongly and significantly increased uPAR/FPR1 co-localization, up to 69% and 61% for uPAR and up to 64% and 76% for FPR1 (Fig. 1A and 1C, first and second set of columns).

uPAR co-localization and association with β1 integrins has been largely demonstrated in the past, as well as their strict collaboration in uPAR cell-signaling [6]–[7], thus we investigated also uPAR/β1 integrin co-localization in the same cells. Indeed, the results showed that, in basal conditions, about 55% of uPAR co-localized with β1 integrins and about 50% of β1 integrins co-localized with uPAR on the cell surface of transfected cells (Fig. 1B and 1C, third and fourth set of columns). Also in this case there was a significant increase of co-localization after serum or W Pep stimulations, up to 70% and 81% for uPAR and up to 70% and 71% for β1 integrins.

These results suggest that uPAR and FPR1 co-localize on the surface of HEK-293 cells to a similar extent as uPAR and β1 integrins; these co-localizations strongly increase after cell stimulation with serum or with a FPR1 ligand, thus supporting the possibility of reciprocal interactions among these three receptors.

### uPAR recruits FPR1 and integrins at the cell surface

**Figure 2:**
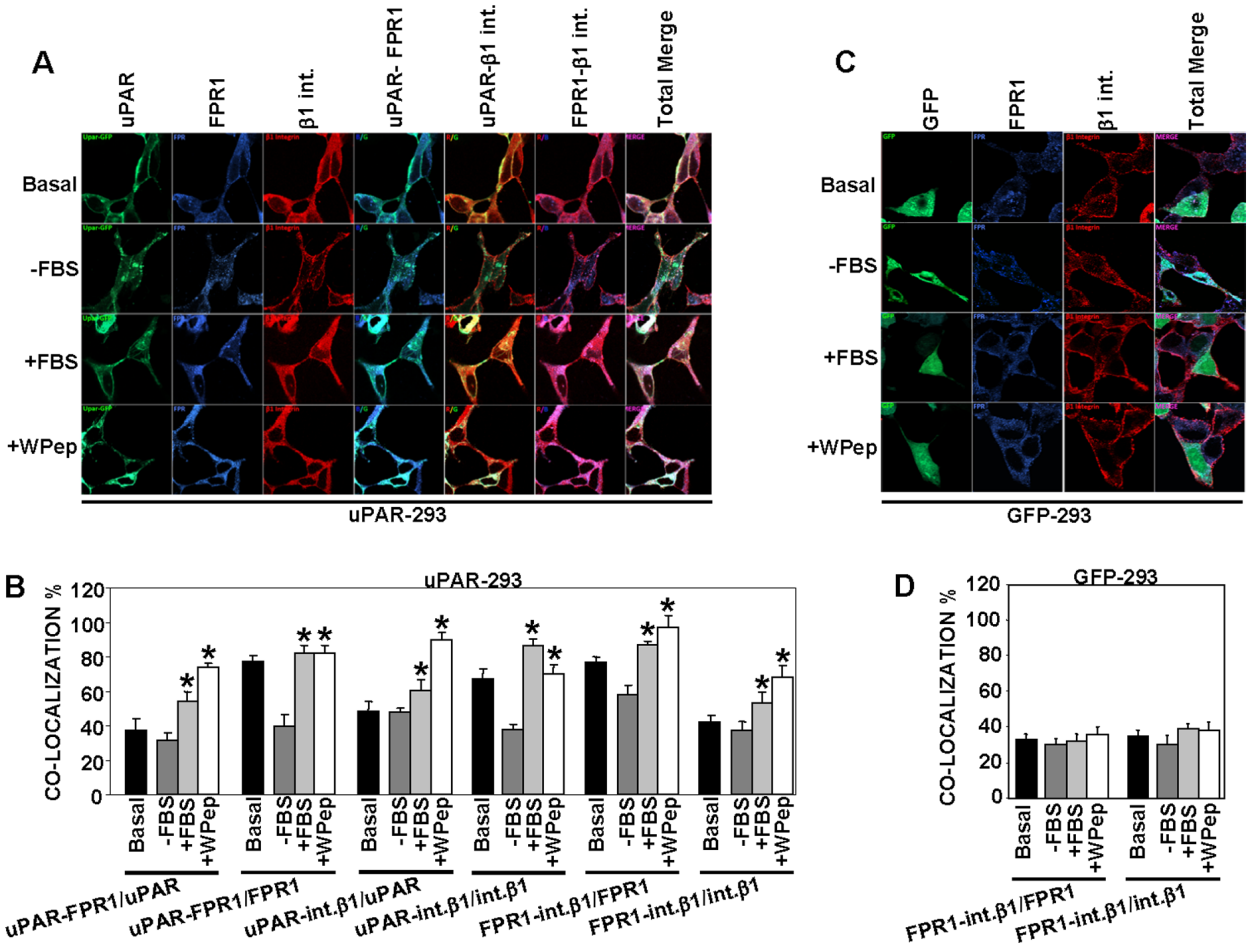
uPAR recruits fMLF receptors and integrins on the cell membrane. **A:** HEK-293 cells were seeded on glass coverslips and transiently transfected with EGFP-uPAR cDNA; after 24 h, cells were incubated for further 24 h in DMEM containing 10% serum (Basal) or in DMEM without serum (−FBS). Prior to fixation, some serum-starved samples were stimulated for 1 h at 37°C, 5% CO2, with 10% FBS in DMEM (+FBS) or with 5 nM WKYMVm peptide (+W Pep) in DMEM. All samples were then fixed, incubated with the anti-FPR1 polyclonal antibody and with the mouse anti-β1 monoclonal antibody, stained with Cy5 or Alexa 594 conjugated secondary antibodies, and analyzed by confocal microscopy. **B:** The degree of co-localization of the fluorescent signals in panel A was quantified on a minimum of 50 different cells by using the LSM 510 software. The number of co-localizing pixels was normalized to the total pixels of each fluorophore. Thus, the number of pixels corresponding to co-localizing uPAR-FPR1 has been normalized to green (uPAR) or blue (FPR1) pixels and reported in 1^st^ and 2^nd^ set of columns, respectively. The number of pixels corresponding to co-localizing uPAR-β1 integrin has been normalized to green (uPAR) or red (β1 Integrin) pixels and reported in 3^rd^ and 4^th^ set of columns, respectively. The number of pixels corresponding to co-localizing FPR1-β1 integrin has been normalized to blue (FPR1) or red (β1 Integrin) pixels and reported in 5^th^ and 6^th^ set of columns, respectively. (*) p≤0.05, as determined by the Student's *t* test. **C:** HEK-293 cells were seeded on glass coverslips and transiently transfected with the empty pEGFP-N1 vector; after 24 h, cells were incubated for further 24 h in DMEM containing 10% serum (Basal) or in DMEM without serum (−FBS). Prior to fixation, some serum-starved samples were stimulated for 1 h at 37°C, 5% CO2, with 10% FBS in DMEM (+FBS) or with 5 nM WKYMVm peptide (+W Pep) in DMEM. All samples were then fixed, incubated with the anti-FPR1 polyclonal antibody and with the rabbit anti-β1 polyclonal antibody, stained with Cy5 or Alexa 594 conjugated secondary antibodies, and analyzed by confocal microscopy. **D:** The degree of co-localization of the fluorescent signals in panel C was quantified on a minimum of 50 different cells by using the LSM 510 software. The number of co-localizing pixels was normalized to the total pixels of each fluorophore. Thus, the number of pixels corresponding to co-localizing FPR1-β1 integrin has been normalized to blue (FPR1) or red (β1 integrin) pixels and reported in the graph.

We then investigated whether uPAR can promote integrin-fMLF-R aggregation on the cell membrane. To this end, we used a different approach for the fluorescence assay, analyzing the localization of a fluorescent protein–tagged uPAR, transiently transfected in HEK-293 cells; FPR1 cDNA was not co-transfected. Then, not only uPAR co-localization with endogenously expressed β1 integrins and FPR1 was examined, but also β1 integrin/FPR1 co-localization. Also with this different approach uPAR/FPR1 and uPAR/β1 integrin co-localization was observed, with a strong and significant increase after serum or W Pep cell-stimulation (Fig. 2A and 2B, left panel, first four sets of columns), confirming previous results (Fig. 1). Indeed, we also evaluated co-localization with FPR1 and β1 integrins of a control molecule, Frizzled-4 (Fz4), a G-protein-coupled receptor not involved in uPA-uPAR system; co-localization values ranged between 20–30% in both cases and did not increase after serum or W Pep cell-stimulation (not shown).

**Figure 3:**
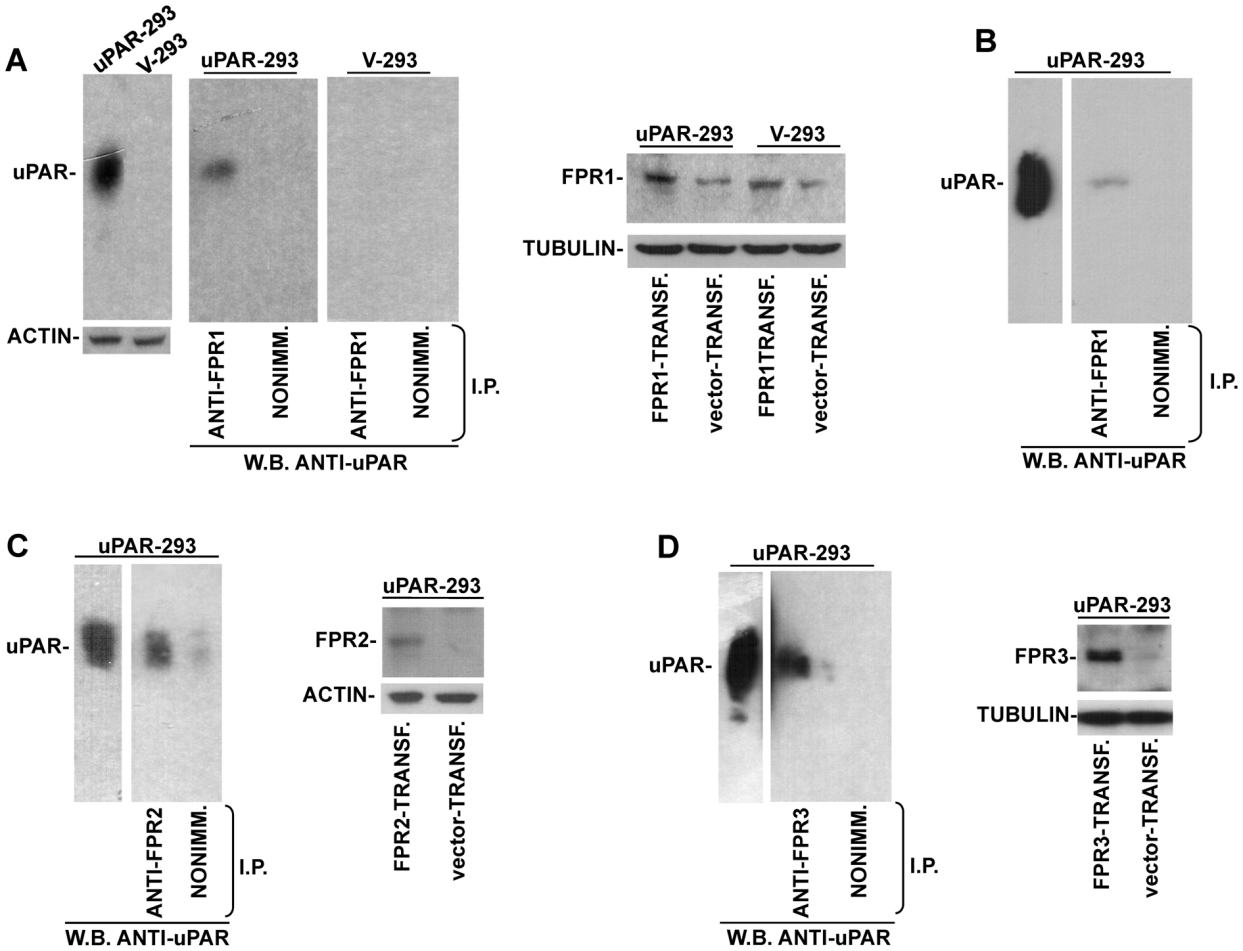
uPAR co-immunoprecipitates with fMLF-Rs. **A:** Lysates of uPAR-293 cells or V-293 cells, transiently transfected with FPR1 cDNA, were analyzed by Western blot with a uPAR-specific monoclonal antibody or immunoprecipitated with anti-FPR1 polyclonal antibodies or nonimmune rabbit immunoglobulins and then analyzed by Western blot with the uPAR-specific monoclonal antibody (left panels). Lysates of FPR1- or vector-transfected uPAR-293 and V-293 cells were analyzed by Western blot with a FPR1-specific polyclonal antibody to assess the levels of FPR1 expression (right panel). Tubulin or actin were detected for loading control. **B:** Lysates of uPAR-293 cells, not transfected with FPR1 cDNA, were analyzed by Western blot with a uPAR-specific monoclonal antibody or immunoprecipitated with anti-FPR1 polyclonal antibodies or nonimmune rabbit immunoglobulins and then analyzed by Western blot with a uPAR-specific monoclonal antibody. **C:** Lysates of uPAR-293 cells, transiently transfected with FPR2 cDNA, were analyzed by Western blot with anti-uPAR antibodies or immunoprecipitated with an anti-FPR2 monoclonal antibody or nonimmune mouse immunoglobulins and analyzed by Western blot with a uPAR-specific polyclonal antibody (left). Lysates of FPR2- or vector- transfected uPAR-293 cells were analyzed by Western blot with anti-FPR2 rabbit or anti-actin mouse antibodies (as loading control) to assess the levels of FPR2 expression (right). **D:** Lysates of uPAR-293 cells, transiently transfected with FPR3 cDNA, were analyzed by Western blot with anti-uPAR antibodies or immunoprecipitated with anti-FPR3 polyclonal antibodies or nonimmune rabbit immunoglobulins and analyzed by Western blot with the uPAR-specific monoclonal antibody (left). Lysates of FPR3- or vector- transfected uPAR-293 cells were analyzed by Western blot with anti-FPR3 rabbit or anti-tubulin mouse antibodies (as loading control) to assess the levels of FPR3 expression (right).

Interestingly, about 77% of FPR1 co-localized with β1 integrins in uPAR-293 cells and about 42% of β1 integrins co-localized with FPR1; their co-localization significantly increased after cell stimulation with serum or with the W Pep (Fig. 2A and 2B, last two sets of columns). β1 integrin/FPR1 co-localization was finally evaluated also in HEK-293 cells transiently transfected with the empty GFP-vector, as a control. Endogenous expression of FPR1 was similar in both uPAR-transfected and vector-transfected control cells, as assessed by Western blot analysis (Fig. 3A). Low values of FPR1/β1 integrin co-localization (about 30%) were observed in uPAR-negative HEK-293 cells; FPR1/β1 integrin co-localization was not increased by serum or W Pep (Fig. 2C and 2D).

These results suggest that uPAR expression on the cell surface promotes FPR1 co-localization with β1 integrins; FPR1/β1 integrin co-localization is strongly increased by serum or the FPR1 ligand only in uPAR-expressing cells.

### uPAR co-immunoprecipitates with fMLF-Rs

Then, biochemical assays were utilized to investigate whether FPR1 associates to uPAR on the cell membrane. FPR1 cDNA was transiently transfected in uPAR-293 cells or in HEK-293 cells stably transfected with the empty vector (V-293 cells), as control. Cells were lysed and immunoprecipitated with a FPR1-specific antibody or with nonimmune immunoglobulins, as control; Western blot analysis of immunoprecipitates with uPAR-specific antibodies showed that uPAR co-immunoprecipitated with FPR1 (Fig. 3A). FPR1 immunoprecipitates were also analyzed by Western blot with anti-β1 integrin antibodies, but, under these experimental conditions, we cannot detect any specific band at the correct Mr (130 kDa) (not shown).

Indeed, FPR1 transfection induced only a moderate increase in FPR1 expression, as shown by Western blot analysis with specific antibodies of transfected cells (Fig. 3A, right panel); thus, we also performed co-immunoprecipitation experiments with uPAR-293 cells not transfected with FPR1, showing that uPAR co-immunoprecipitates also with endogenous FPR1 (Fig. 3B).

To investigate whether uPAR associates also to the other fMLF-Rs, uPAR-293 cells were transiently transfected with FPR2 and FPR3 cDNAs, which are not or poorly expressed by HEK-293 cells [15], and the corresponding lysates were immunoprecipitated with FPR2- and FPR3- specific antibodies. Western blot analysis with uPAR-specific antibodies of the immunoprecipitates showed, also in this case, a specific band corresponding to uPAR (Fig. 3C and 3D).

Co-immunoprecipitation assays thus showed that uPAR associates to all three fMLF-Rs. Since uPAR associates also to integrins [7], it is reasonable to hypothesize that uPAR could bridge both molecules at the cell surface.

### uPAR expression controls cell migration toward serum

uPAR expression is required for cell migration toward fMLF and, conversely, fMLF receptors are required for cell migration toward uPA, suggesting a functional interaction between these receptors [2]. However, cell-surface uPAR regulates also migration toward other ligands, such as SDF1 and PDGF, and regulates the activity of the EGFR [9]; the involvement of fMLF-Rs has been investigated and demonstrated only in the SDF1-dependent migration [15].

We hypothesized that uPAR could be able to regulate cell migration independently of a specific chemoattractant, through a mechanism involving fMLF-Rs and integrins, as shown in the SDF1-dependent migration. To test our hypothesis we evaluated the migration of uPAR-293 and V-293 control cells toward serum (which could be considered a mixture of various chemoattractants), blocking or not uPAR interactions at the cell surface. Migration assays were performed on filters coated with collagen (CG), which should not have any type of interaction with uPAR and thus should not interfere with uPAR-dependent cell migration.

**Figure 4:**
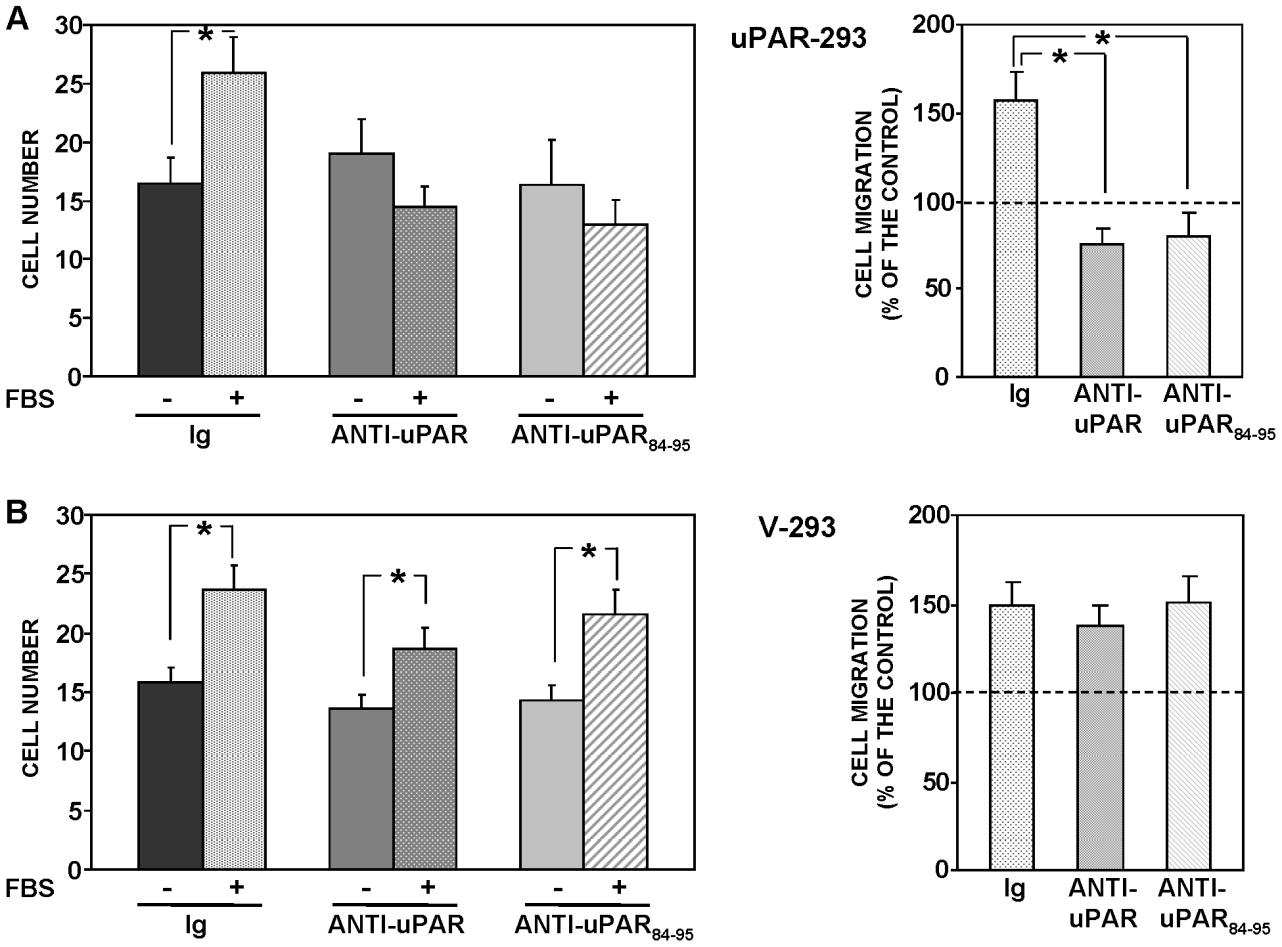
uPAR expression controls cell migration toward serum. uPAR-293 cells (**A**) or V-293 cells (**B**) were pre-incubated with nonimmune immunoglobulins (Ig), anti-uPAR or anti-uPAR_84–95_ polyclonal antibodies, plated in Boyden chambers and allowed to migrate toward 10% FBS. Migrated cells were fixed, stained with hematoxylin, and counted (left panels). The values are the mean±SD of three experiments performed in triplicate. (*) p≤0.05, as determined by the Student's *t* test. Results of migration assays are also expressed as percentage of cells migrated towards serum over the cells migrated without serum; 100% values represent cell migration in the absence of chemoattractants (right panels). (*) p≤0.05, as determined by the Student's *t* test.

uPAR-293 cells or V-293 cells were allowed to migrate toward serum in the presence of non-immune immunoglobulins or of polyclonal antibodies directed against the whole molecule of uPAR or against the uPAR_84–95_ region (residues 84–95 of uPAR), the latter corresponding to the region of the soluble form of cleaved uPAR involved in the binding to fMLF-Rs [10], [24]. Both uPAR-293 cells and V-293 control cells efficiently migrated toward serum; anti-uPAR antibodies did not exert any effect on their basal migration (in the absence of chemoattractant) (Fig. 4A and 4B, left panels). However, anti-uPAR antibodies totally inhibited serum-induced migration of uPAR-293 cells, without exerting any effect on serum-induced migration of V-293 control cells (Fig. 4A and 4B).

These results suggest that even if HEK-293 cells are able to migrate independently of uPAR expression, when they express uPAR, their migration seems to become totally and irreversibly uPAR-dependent, in fact it is completely inhibited by uPAR blocking.

### fMLF receptors and β1 integrins are involved in uPAR capability to control cell migration

Serum-induced uPAR-dependent cell migration was inhibited by a polyclonal antibody directed against the whole uPAR molecule which, presumably, blocks all uPAR interactions at the cell-surface, and by an antibody recognizing the uPAR region involved in the interaction with fMLF-Rs (Fig. 4). We then investigated whether fMLF-Rs and/or integrins, which interact with uPAR, are involved in uPAR capability to control cell migration.

**Figure 5:**
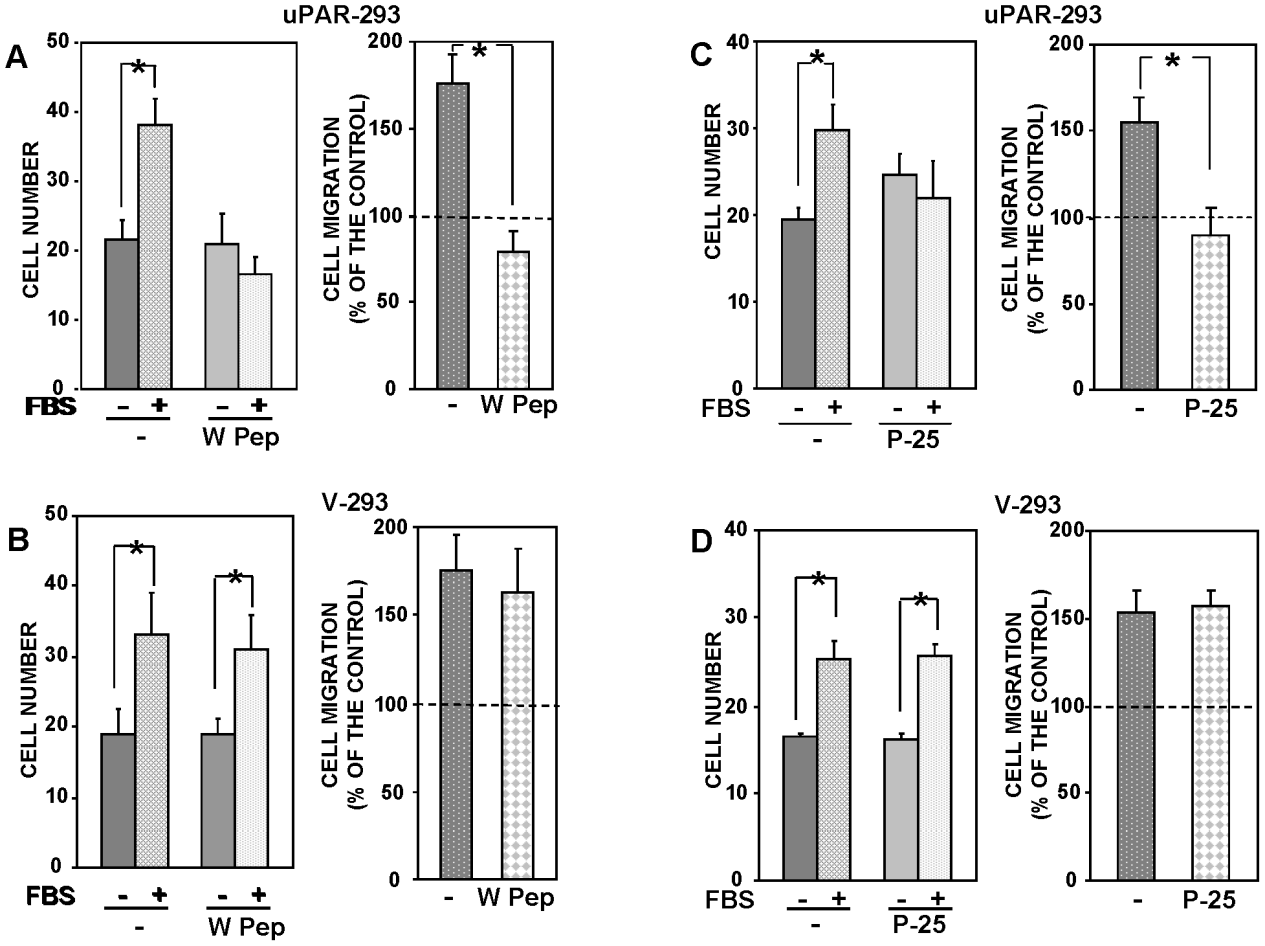
fMLF receptors and β1 integrins are involved in uPAR capability to control cell migration. uPAR-293 cells (**A and C**) or V-293 cells (**B and D**) were pre-incubated with diluent (-) or W Peptide (W Pep) (**A and B**), or with diluent (-) or P-25 peptide (**C and D**). Cells were then plated in Boyden chambers and allowed to migrate toward 10% FBS. Migrated cells were fixed, stained with hematoxylin, and counted (left panels). The values are the mean±SD of three experiments performed in triplicate. (*) p≤0.05, as determined by the Student's *t* test. Results of migration assays are also expressed as percentage of cells migrated towards serum over the cells migrated without serum; 100% values represent cell migration in the absence of chemoattractants (right panels). (*) p≤0.05, as determined by the Student's *t* test.

fMLF receptors can be desensitized by pre-treating cells with their ligands before migration [11]. We then performed migration assays with uPAR-293 cells and V-293 cells after pre-incubation with or without W Pep, a ligand of fMLF-Rs. Desensitization of fMLF-Rs did not affect basal migration of both uPAR-293 and V-293 cells (Fig. 5A and 5B, left panels) but totally impaired uPAR-293 cell migration toward serum without exerting any significant effect on serum-induced migration of V-293 control cells (Fig. 5A and 5B).

We then investigated whether uPAR capability to control cell migration could depend also on its interaction with integrins. To this end, migration assays were performed in the presence or in the absence of the P-25 peptide, which has been shown to disrupt uPAR interactions with β1 or β2 integrins [17]. Treatment of uPAR-293 cells with the P-25 peptide inhibited their migration toward serum, whereas treatment of V-293 control cells with the same peptide did not exert any effect (Fig. 5C and 5D). Also in this case basal migration of both cell types was not affected (Fig. 5C and 5D, left panels).

Altogether, these results suggest that, when expressed, uPAR takes control of cell migration by interacting with β1 integrins and fMLF receptors.

### uPAR-dependent cell migration is mediated by signaling mediators not involved in uPAR-independent cell migration

We then investigated whether uPAR-dependent and uPAR-independent cell migrations toward serum were mediated by same signaling pathways.

**Figure 6:**
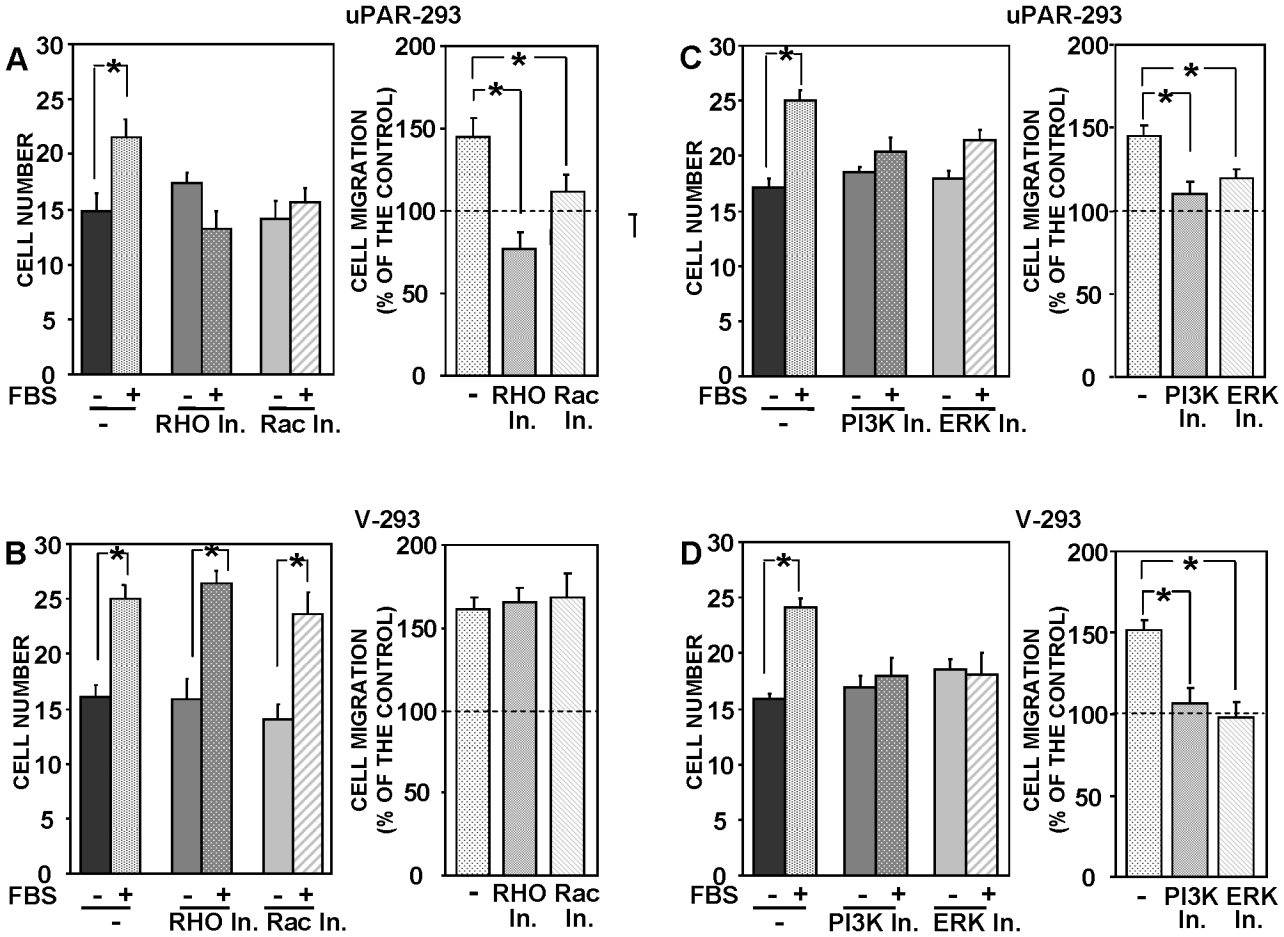
uPAR-dependent cell migration is mediated by signaling mediators not involved in uPAR-independent cell migration. uPAR-293 cells (**A and C**) or V-293 cells (**B and D**) were pre-incubated with diluents (-) or inhibitors of Rho- or Rac1-dependent signaling pathways (**A and B**), or with diluents (-) or inhibitors of PI3K or ERK-MAPKs (**C and D**). Cells were then plated in Boyden chambers and allowed to migrate toward 10% FBS. Migrated cells were fixed, stained with hematoxylin, and counted (left panels). The values are the mean±SD of three experiments performed in triplicate. (*) p≤0.05, as determined by the Student's *t* test. Results of migration assays are also expressed as percentage of cells migrated towards serum over the cells migrated without serum; 100% values represent cell migration in the absence of chemoattractants (right panels). (*) p≤0.05, as determined by the Student's *t* test.

It has been previously shown that small GTPases as Rac1, RhoA, Cdc42 and RhoB mediate uPA- induced cell migration [6], [25], we thus performed migration assays with uPAR-293 cells and V-293 control cells in the presence or in the absence of inhibitors of the Rac-specific GEF (guanine nucleotide exchange factor) Trio and Tiam1 and of the Rho-associated kinase (ROCK). We found Rac1 and Rho involvement in uPAR-controlled migration, since inhibition of their downstream signaling pathways significantly impaired migration toward serum of uPAR-expressing cells (Fig. 6A). By contrast, both mediators were not involved in serum-induced migration of uPAR-negative cells (Fig. 6B).

uPAR-dependent signaling pathways can also include PI3K and lead to the activation of ERK MAPKs [6]. Indeed, specific inhibitors for PI3K and ERKs inhibited serum-induced migration of both uPAR-293 cells and V-293 control cells, indicating that these two mediators are used by both uPAR-expressing and uPAR-negative cells for migration (Fig. 6C and 6D, respectively).

These results suggest that uPAR-controlled migration, which is allowed by uPAR interactions with fMLF-Rs and β1 integrins, involves specifically small Rho GTPases as signaling mediators.

### uPAR expression controls cell migration toward EGF

All previous migration assays were performed using serum as chemoattractant, which, indeed, is a mixture of various chemoattractants, including also uPAR ligands, such as uPA or VN. Thus, it was possible to hypothesize that, in uPAR-expressing cells, the effect of uPAR ligands prevailed on that of other serum chemoattractants, inducing activation of new signaling pathways beside the ones activated in V-293 control cells. We then performed migration assays using purified EGF as chemoattractant. EGF is a growth factor largely present in serum and does not bind uPAR, even if its receptor, EGFR, is involved in uPAR signaling [26].

**Figure 7:**
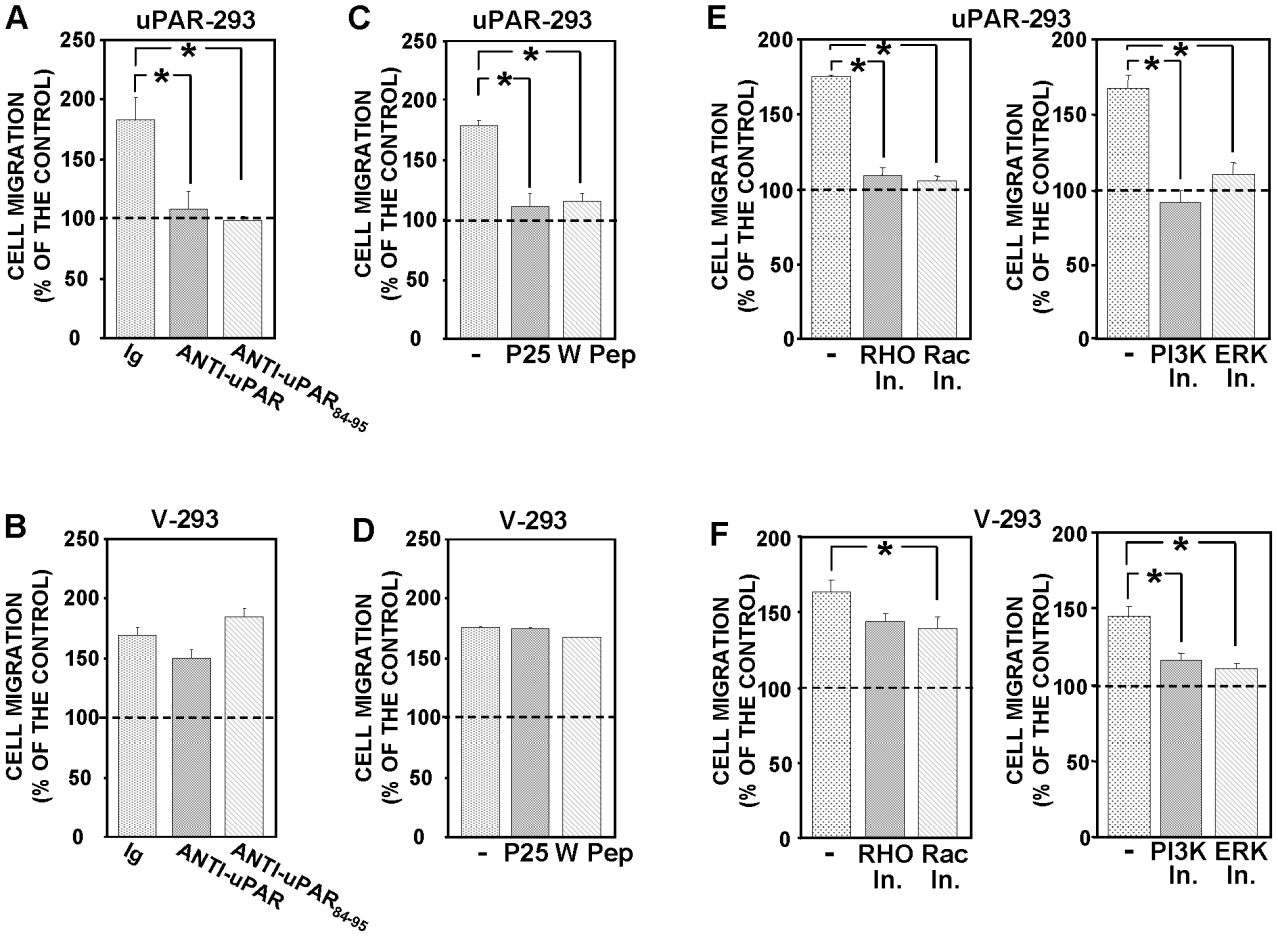
uPAR expression controls cell migration toward EGF. Stably-transfected uPAR-293 cells (**A, C, E**) or V-293 cells (**B, D, F**) were pre-incubated with nonimmune Ig (**-**) or anti-uPAR or anti-uPAR_84–95_ polyclonal antibodies (**A and B**), with diluents (-) or P-25 or W (W Pep) peptides (**C and D**), with diluents (-) or inhibitors of Rho- or Rac1-dependent signaling pathways (**E and F, left panels**), with diluents (-) or inhibitors of PI3K or ERK-MAPKs (**E and F, right panels**). Cells were then plated in Boyden chambers and allowed to migrate toward 100 ng/ml EGF. Migrated cells were fixed, stained with hematoxylin, and counted; results are expressed as percentage of cells migrated towards EGF over the cells migrated without EGF; 100% values represent cell migration in the absence of chemoattractants. The values are the mean±SD of three experiments performed in triplicate. (*) p≤0.05, as determined by the Student's *t* test.

EGF induced migration of both uPAR-293 and V-293 cells with a similar efficiency (Fig. 7). We then assessed whether uPAR expression and/or interactions influenced HEK-293 cell migration toward EGF, as it occurs in cell migration toward serum. Indeed, polyclonal antibodies against the whole uPAR molecule or the uPAR_84–95_ region, P-25 peptide and fMLF-R desensitization significantly inhibited EGF-induced migration of uPAR-293 cells (Fig. 7A and 7C), without affecting basal migration (not shown), whereas they did not exert any significant effect on EGF-induced migration of V-293 control cells (Fig. 7B and 7D).

Then, we evaluated the effect of signaling inhibitors on EGF-induced migration of uPAR-293 and V-293 cells, showing that, also in this case, migration of uPAR-expressing cells involved both Rho and Rac1 small GTPases, unlike migration of uPAR-negative cells, even a very low but significant effect was observed with the Rac-1 inhibitor also in V-293 cells (Fig. 7E and 7F, respectively).

Thus, the results obtained in chemotaxis assays using a purified chemoattractant are very similar to those obtained in chemotaxis assays using serum as chemoattractant, suggesting that uPAR controls the migration mechanism of the cell rather than the migration toward specific factor/s.

### uPAR depletion or blocking impairs migration of uPAR-expressing cells

**Figure 8:**
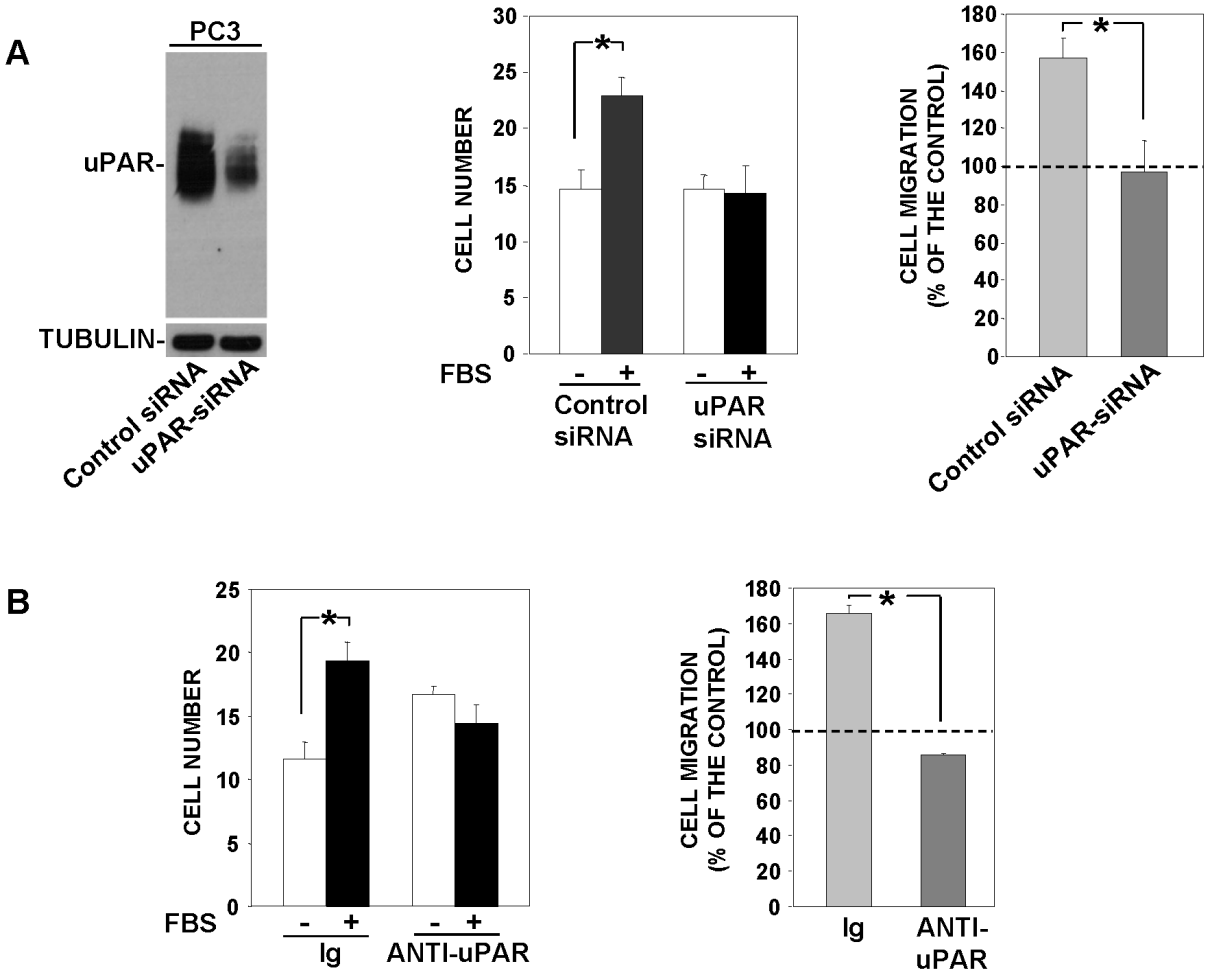
uPAR depletion or blocking impairs migration of uPAR-expressing cells. **A:** Prostate carcinoma (PC3) cells were transfected with a uPAR-targeting siRNA or a non-targeting control siRNA; then, cells were partly lysed for Western blot analysis with a uPAR-specific antibody (left) and partly loaded in Boyden chamber and allowed to migrate toward 10% FBS. Migrated cells were fixed, stained with hematoxylin, and counted (middle panel). The values are the mean±SD of a representative experiment performed in triplicate. Results of the migration assay are also expressed as percentage of cells migrated towards serum over the cells migrated without serum; 100% values represent cell migration in the absence of chemoattractant (right). (*) p≤0.05, as determined by the Student's *t* test. **B:** PC3 cells were pre-incubated with nonimmune immunoglobulins (Ig) or anti-uPAR polyclonal antibodies, plated in Boyden chambers and allowed to migrate toward 10% FBS. Migrated cells were fixed, stained with hematoxylin, and counted (left). Results of the migration assay are also expressed as percentage of cells migrated towards serum over the cells migrated without serum; 100% values represent cell migration in the absence of chemoattractant (right). The values are the mean±SD of three experiments. (*) p≤0.05, as determined by the Student's *t* test.

Since uPAR over-expression in uPAR-negative cells takes control of their migration, we assessed whether uPAR depletion or blocking in cells which constitutively express uPAR, as prostate carcinoma (PC3) cells [25], impair their migration. PC3 cells were transfected with a uPAR-targeting siRNA or a control siRNA, then, cells were partly lysed for Western blot analysis and partly used for chemotaxis assays. Western blot analysis with uPAR-specific antibodies of transfected-cell lysates showed that control cells expressed high uPAR levels, which were strongly reduced in PC3 cells transfected with the uPAR-targeting siRNA (Fig. 8A, left); uPAR-depletion impaired PC3 cell migration toward serum (Fig. 8A, right). Accordingly, PC3 cell migration toward serum was completely blocked by anti-uPAR polyclonal antibodies (Fig. 8B).

These results suggest that uPAR takes control of cell migration also in cells which constitutively express it; in fact inhibition of its expression or interactions abrogate cell ability to migrate.

## Discussion

In the last decade we and others have observed a role for the fMLF-Rs in uPAR activities. We started from the finding of Blasi's group that the cleaved form of soluble uPAR, exposing the residues 88–92 at its N-terminus, is a ligand for the low-affinity receptor for fMLF (FPR2) and is able to activate it, thus inducing cell migration [10], [24]. We subsequently observed that the peptide covering this uPAR region (uPAR_84–95_) was able to induce cell migration by stimulating also the other two fMLF-Rs, FPR1 and FPR3 [27]–[28].

fMLF-Rs also cross-talk with cell-surface uPAR. In fact, we and others showed that fMLF-induced cell migration requires cell-surface uPAR expression [18], [20]–[21]; on the other hand, uPA-induced cell migration requires not only cell-surface uPAR expression but also fMLF-R expression [10]. Cell-surface uPAR exerts its regulatory effect on the fMLF-induced migration both in the cleaved form exposing the uPAR_84–95_ region and in the full-length form, which, unlike full-length suPAR, exposes this specific region [12], [18]. Further, it has been reported that mutations in the uPAR_84–95_ region of cell-surface uPAR interfere with uPA-dependent signals and regulate FPR1 activation [21], [29].

Altogether, these findings strongly suggested an interaction between cell-surface uPAR and fMLF-Rs, nevertheless, their association has never been explored in detail. We now show that, in uPAR-negative HEK-293 cells stably transfected with uPAR-cDNA, or transiently transfected with an EGFP-uPAR cDNA, a fraction of uPAR co-localizes with FPR1 and, as expected, with β1 integrins, and viceversa, a fraction of FPR1 and β1 integrins co-localizes with uPAR. Interestingly, cell stimulation with a generic stimulus as serum, or with a specific stimulus as a fMLF-R ligand, strongly promotes not only uPAR/β1 integrin and uPAR/FPR1 co-localizations, but also co-localization of FPR1 with β1 integrins. These observations suggest that uPAR is able to recruit a large fraction of FPR1 and β1 integrins and strongly promotes their co-localization when cells are stimulated, suggesting a functional meaning for these events occurring at the cell surface.

Indeed, uPAR-FPR1 closeness allows their co-immunoprecipitation; uPAR co-immunoprecipitates also with FPR2 and FPR3, in agreement with the fact that cell-surface uPAR functionally interacts with all three fMLF-Rs and that cleaved suPAR activates all of them [2], [9]. Since it has been largely demonstrated that uPAR contains also binding sites for β1 integrins, not localized in the uPAR _84–95_ region [30]–[31], it is reasonable to hypothesize that uPAR contemporaneously associates to both these molecules, as suggested by fluorescence assays, thus bridging them at the cell surface.

We then explore the functional meaning of these specific uPAR interactions. We previously demonstrated that uPAR is able to regulate the activity of CXCR4, the receptor for the SDF1 chemokine, through a mechanism involving integrins and fMLF-Rs [15]. We now investigate whether this uPAR regulatory capability is restricted to CXCR4 or relies on a general mechanism, involving integrins and fMLF-Rs, by which uPAR can regulate cell migration, independently of the specific chemoattractant. To this end, we performed cell migration assays with uPAR-expressing and uPAR-negative HEK-293 cells, using serum as chemoattractant, which is a mixture of various chemoattractants, and observed the effect of the impairment of uPAR interactions on cell migration. uPAR-negative cells migrated toward serum with a similar efficiency as compared to uPAR-expressing cells. Nevertheless, uPAR expression rendered cell migration totally uPAR-dependent, since blocking uPAR interactions blocked uPAR-293 cell migration, unlike migration of uPAR-negative control cells. This conclusion was confirmed in prostate carcinoma (PC3) cells which constitutively express uPAR; in fact, uPAR depletion by a uPAR-specific siRNA or uPAR blocking by a uPAR-specific antibody completely impaired PC3 cell migration toward serum.

Analysis of signaling mediators involved in cell migration showed the selective involvement of Rho and Rac-1 small GTPases in serum-induced migration of uPAR-293 cells, suggesting that, when formed, the uPAR/FPR1/integrin complex activates new signaling pathways, thus probably taking control of the migration process.

However, we reasoned that the involvement of new signaling mediators in uPAR-293 cells could be merely due to the specific uPAR stimulation by a uPAR ligand present in serum, such as uPA or VN. Thus, we repeated all migration experiments with a purified chemoattractant, EGF, which does not bind uPAR, is a serum component and a suitable chemoattractant for epithelial cells. EGF induced migration of both uPAR-293 and V-293 cells; treatments blocking uPAR interactions at the cell surface impaired EGF-induced migration of uPAR-expressing cells without interfering with migration of uPAR-negative cells; uPAR-293 cell migration involved all tested signaling mediators, unlike V-293 cell migration. Thus, all results obtained with a purified serum chemoattractant were comparable to those obtained with total serum. We have to underline that uPAR has been shown to associate and to activate the EGFR, which has been proposed as a possible component of the uPAR-signaling machinery [26]. Nevertheless, more recent reports showed transactivation of the EGFR by FPR1 in glioblastoma cells and in monocytes, in which FPR1 modulates the activation of EGFR and TrkA, the NGF receptor [32]–[33]; thus, we cannot exclude that the uPAR-mediated activation of EGFR observed in previous studies [26] involved also FPR1.

Indeed, integrins can directly activate growth factor receptors in the absence of any growth factor ligand [34]; in fact, a hierarchy is established where cell adhesion, which induces integrin clustering, represents the limiting factor - and an alternative priming event - for growth factor receptor activation. Specifically, the tyrosine kinase receptors for EGF, PDGFβ, VEGF, hepatocyte growth factor (HGF), and macrophage-stimulating protein (MSP) are all transactivated after integrin engagement [35]. The simple integrin clustering can regulate integrin activity, as well as inside-out signaling, when external stimuli initiate intracellular signals that alter the affinity state of the integrins [36]. In fact, fMLF-Rs activation can regulate the activity of various integrins [37]–[39].

**Figure 9:**
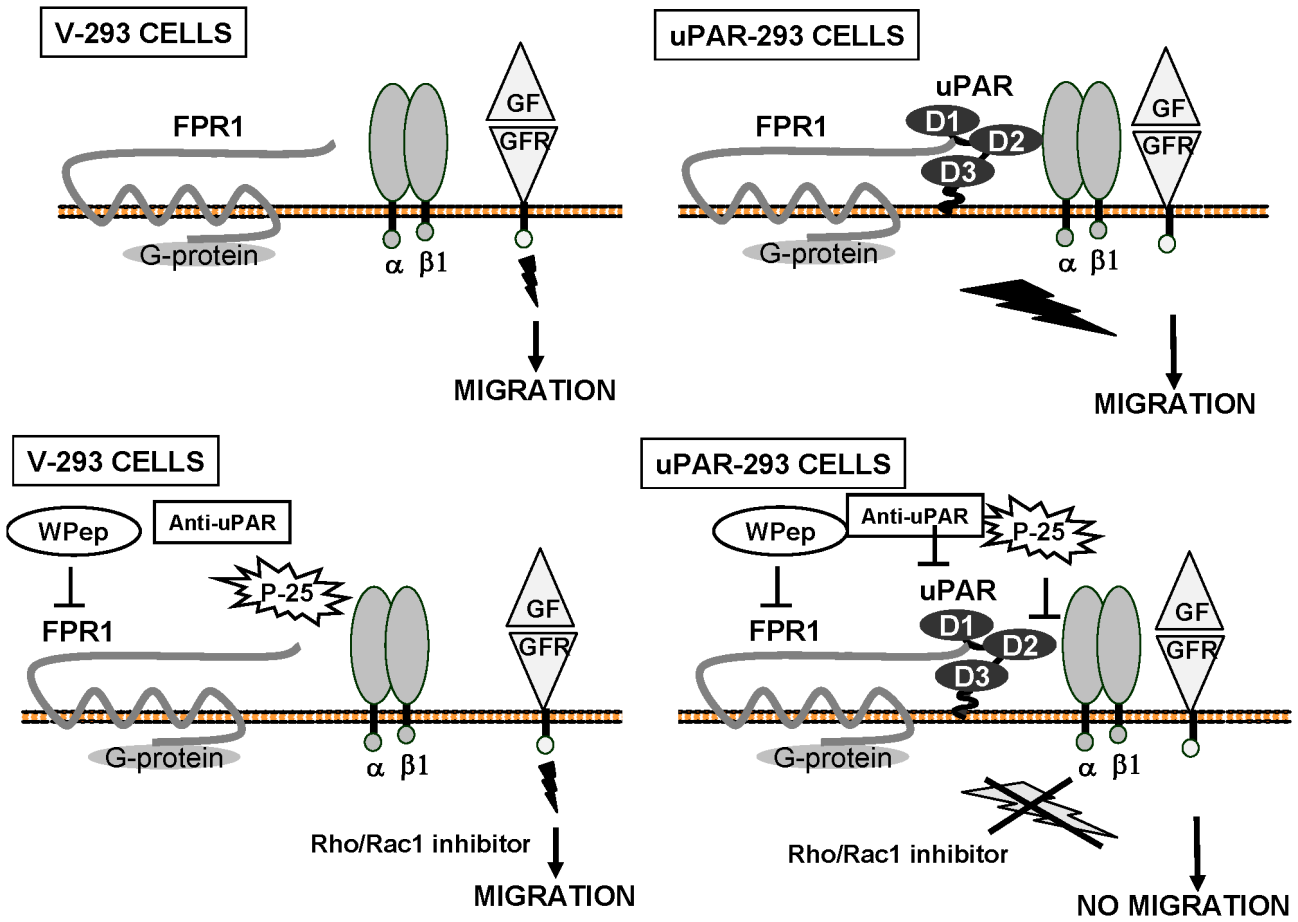
uPAR expression controls cell migration. uPAR-293 and V-293 cells efficiently migrate toward serum growth factors or EGF (GF). However, uPAR, when expressed, recruits and bridges fMLFRs and β1 integrin at the cell surface, thus driving pro-migratory signaling (upper panels). In fact, anti-uPAR antibodies (anti-uPAR), FPR1 desensitization by the W peptide (W Pep), inhibition of uPAR/β1 integrin interaction by P-25 peptide (P-25) or of specific cell signalling mediators block migration of uPAR-293 cells without affecting migration of uPAR-negative V-293 control cells (lower panels).

In this context, our results, depicted in Fig. 9, suggest that uPAR, which can associate to β1 integrins and fMLF-Rs (ref.7 and Fig. 3), could act as a docking cell-surface molecule for both receptor types, recruiting and bridging them on the cell surface. Thus, cell-surface uPAR could bind and activate fMLF-Rs, as the cleaved soluble form of uPAR is able to do [10]; stimulated fMLF-Rs, in turn, could activate signaling pathways able to modulate activation status and/or signaling of uPAR-recruited β1-integrins, which are crucial for the activity of various growth-factor receptors [33]–[36]. It is noteworthy that a fraction of uPAR is associated to lipid rafts, which are cholesterol-rich membrane platform concentrating signaling mediators [40].

In conclusion, we propose that uPAR overexpression controls the mechanisms of cell directional migration by recruiting integrins and FPR1 at the cell surface and regulating their signaling pathways.
